# Retained surgical item (Gossypiboma): a case report and literature review

**DOI:** 10.1097/MS9.0000000000000992

**Published:** 2023-06-17

**Authors:** Mohammed A. Alsuhaimi, Hallah S. Alghamdi, Sarah A. Alshaiji, Mohammed A. Fayi, Sahar M. Aldhafeeri

**Affiliations:** Dammam Medical Complex, Dammam, Saudi Arabia

**Keywords:** gossypiboma, intestinal obstruction, intra-abdominal, retained surgical item, surgical complication

## Abstract

Retained surgical item is a known incidental surgical complication that is a mass of a sponge. This cotton matrix is left behind in the body cavity after surgical procedures. It is an incidental, rare medical error.

**Case presentation:**

Here, the authors present a case of a 30-year-old woman who had undergone a cesarean section 2 months ago and presented with the cardinal symptoms of small bowel obstruction. A computerized abdominal tomography (CT) scan showed a well-defined tubular hyperdense structure adherent to the anterior abdominal wall that exerts a mass effect on the adjacent small bowel loops. Following the computerized abdominal tomography results, an exploratory laparotomy resection and anastomosis of a small segment of the ileum were performed. The postoperative period was uneventful, and the patient has remained disease-free to date.

**Clinical discussion:**

Because it is not anticipated, and its clinical manifestations are variable, it is frequently misdiagnosed, and often unnecessary radical surgical procedures are performed.

**Conclusion:**

It should be considered in the differential diagnosis of any postoperative case with an unresolved or unusual presentation.

## Introduction and importance

Highlights

- Diagnosing a gossypiboma can be challenging, as symptoms may not appear until weeks or even years after the surgery.
- Medical errors, such as miscounting surgical sponges, poor documentation, or inadequate communication among surgical team members.
- These retained sponges can cause a variety of early and late complications.

The first reported case of gossypiboma was documented in 1884 by a British surgeon named Wilson. Wilson reported a case of a patient who underwent surgery for a fibroid tumor and later developed symptoms of abdominal pain and fever, only to discover that a surgical sponge had been left inside the patient’s body during the initial surgery^1^. Since then, numerous cases of gossypiboma have been reported in medical literature, and the condition has become recognized as a rare but potentially serious complication of surgical procedures. The work has been reported in line with the Surgical CAse REport (SCARE) 2020 criteria^2^.

## Case presentation

**Figure 1:**
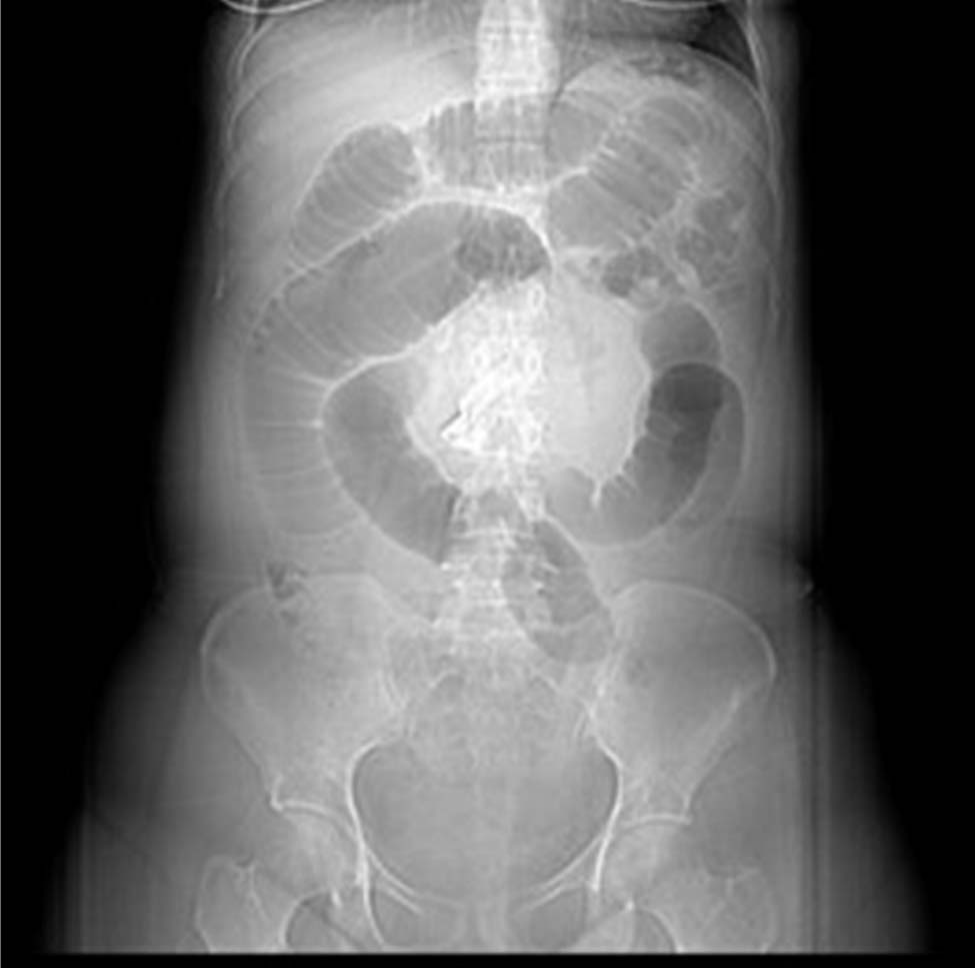
The abdominal radiograph showed dilated small bowel loops.

**Figure 2:**
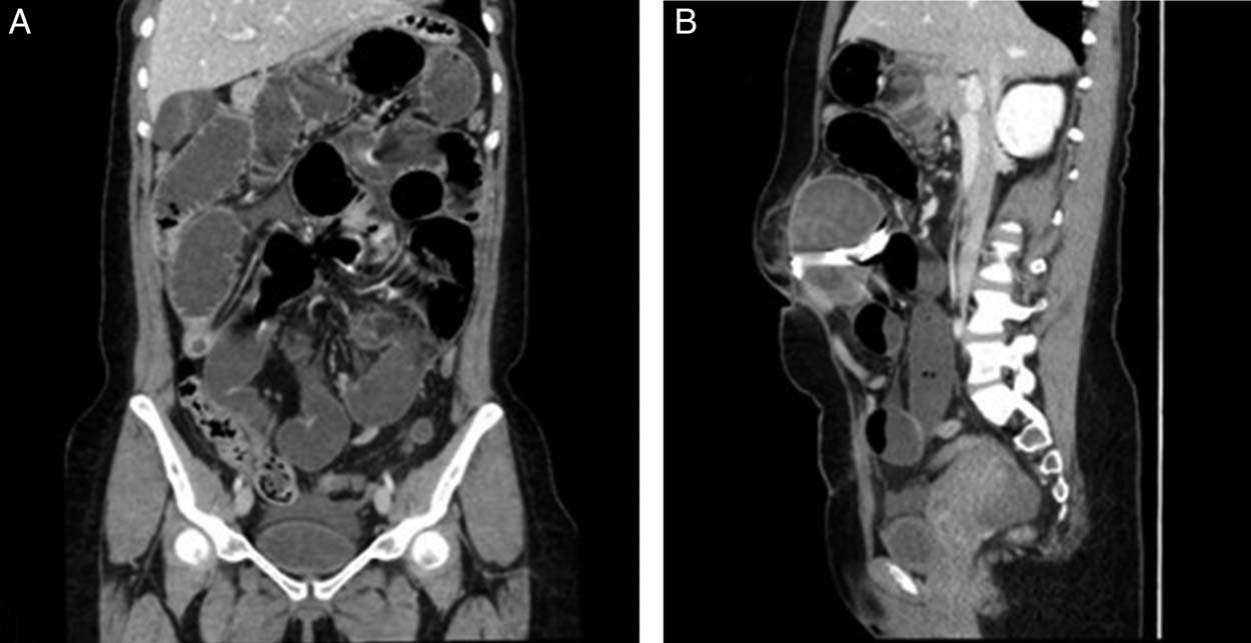
Findings were suggestive of partial small bowel obstruction.

**Figure 3:**
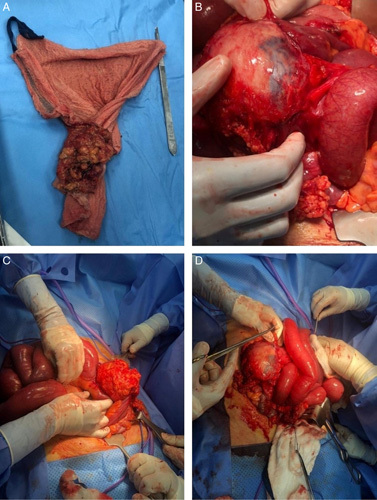
Adhesiolysis was done to remove the granuloma, with resection and anastomosis of a small segment of the ileum. The content of the granuloma was a large abdominal surgical towel from the patient’s previous surgery.

A 30-year-old woman, medically free, who had undergone a cesarean section (C-section) 2 months back in the other hospital. The patient presented to the emergency department complaining of continuous abdominal pain in the umbilical region for 2 days. The pain was associated with nausea, vomiting, and constipation and started about 2 months ago. Family, drug, and psychological history were unremarkable. Upon examination, the patient was lying on the bed in severe pain, and she was alert, conscious, and oriented. Her heart rate was 120/min, blood pressure was 113/74 mmHg, respiratory rate was 20 breaths/min, and temperature was 36.9°C. Upon inspection of the abdomen, a Pfannenstiel incision scar and abdominal distension were noted; upon palpation, the abdomen was soft and lax, along with periumbilical swelling. Laboratory workups revealed leucocytosis of 16.25×10^9^/l, otherwise unremarkable results. For imaging, the abdominal radiograph showed dilated small bowel loops (Fig. 1). A Computerized abdominal tomography (CT) scan showed a well-defined tubular hyperdense structure adherent to the anterior abdominal wall. It was anterolateral to the umbilical region that is exerting a mass effect on the adjacent small bowel loops. It was associated with well-defined heterogeneous wall enhancement and fluid collection. The jejunum and proximal ileal loops showed dilatation reaching a maximum diameter of 5.2 cm, and are fluid-filled with some focalization and multiple air-fluid levels. Subsequent collapsed distal ileal loops were noticed as well, suggesting an area of the transition zone. The transverse colon, descending colon, sigmoid, and rectum all collapsed. Furthermore, the findings were suggestive of partial small bowel obstruction (Fig. 2). Following the CT results, an exploratory laparotomy revealed a granuloma at the connection of the small and large bowels with pus collection. Adhesiolysis was done to remove the granuloma, with resection and anastomosis of a small segment of the ileum. The content of the granuloma was a large abdominal surgical towel from the patient’s previous surgery. The patient was recovering well postoperatively; a complete diet was started on day five postoperation, and she was fit for discharge in good, stable condition on the 7th day. (Fig. 3). The patient was followed up as an outpatient and rested asymptomatically before resuming her life activities.

## Clinical discussion

Gossypiboma is a nonmedical term used to describe a mass of cotton sponge that is retained by mistake in the patient’s body cavity and also can be defined as any item used during surgery, such as instruments, sponges, or towels/pads, that was retained unintentionally inside the body cavity in the operating room. More specifically, when the retained object is made out of cotton, usually a towel or surgical gauze, it is called Gossypiboma, where ‘gossypium’ in Latin means cotton, and the word ‘boma’ means place of concealment^3^. It’s considered the most common retained surgical item (RSI), RSI is a never event, meaning that it should never happen^4^. It is preventable, and by applying proactive measures based on causation only, we can avoid causing harm to the patients. This dilemma has been described by several terms in medical literature, such as retained surgical sponges, textilomas, or cottonoid syndrome. Studies suggested that the incidence of Gossypiboma following abdominal surgery is estimated to be between 1 and 1000 to 1 and 1500 procedures. However, the actual number of cases may be higher due to underreporting, possibly due to associated medicolegal issues. RSI, including gossypibomas, were found to be the third most common cause of malpractice in a retrospective study of surgical malpractice conducted in the United States. The reported incidence in the abdominal cavity (56%), pelvis (18%), and thorax (11%). Its worth to highlighted there are several risk factors; the most significant risk factors were any emergency procedures, unplanned changes in the surgery, the patient’s high BMI, and lack of supervision by a senior surgeon^2,5,6^. In the current case, we are reporting a case of partial small bowel obstruction secondary to intrabdominal Gossypiboma at the anterior abdominal wall, which was anterolateral to the umbilical region that exerted a mass effect that had presented 2 months following a cesarean section as for categorizing the operation, a cesarean section is generally considered an abdominal procedure, since it involves incisions through the abdominal wall and peritoneum. Some might also call it a pelviabdominal procedure involving both the abdominal and pelvic regions. However, it would not be accurate to call it a ‘pure’ pelvic operation, as the surgery directly involves the abdominal cavity. We need to highlight that In a typical cesarean section, the peritoneum is indeed opened. This is necessary to access the uterus and deliver the baby. However, surgeons take care to minimize adhesions (scar tissue) that can form as a result of surgery, which could potentially lead to adhesion and can be got adherent to small bowels subsequently, lead to adhesion and this gotten adherent to the RSI (abdominal towel) that is unintentionally left behind at the end of the surgery. Clinically, it is challenging to be diagnosed and most of the patients present with classical cardinal symptoms and signs of any bowel obstruction or an RSI-associated complication (Table 1), most of the time due to its nonspecific clinical manifestation, it can be misdiagnosed as an abdominal tumor or abscess and umbilical hernia^7^. General speaking; there is two pathophysiology aspect of textilomas that reflected into its clinical patterns: the exudative and fibrinous textilomas. Exudative Gossypiboma is an inflammatory reaction in response to a foreign body; it can be complicated by abscess formation, fistulas, or sepsis. Whereas fibrinous Gossypiboma is when the retained foreign body is encapsulated with scar tissue that eventually forms granuloma, which can migrate to the bowel, causing obstruction, as in our case. Unlike exudative, fibrinous is a late presentation ~60 days after a foreign body has been retained^8–11^. The main diagnostic tools are CT scans and MRIs. The diagnostic radiographic features of Gossypiboma in CT scan include spongiform appearance, low-density mass with a thin enhancing capsule, and deposition of calcifications^12–14^. American College of Surgeons’ recommendations regarding preventing unintentionally RSI. Good communication among perioperative staff, proper surgical count, adequate wound exploration before surgical closure, and use of items can reduce the incidence^15,16^. If there is doubt, then imaging should be performed. None of these prevention recommendations are reliable when used alone. Overall, the main prevention of RSI is the counting technique, which has a high error probability and limitations, especially in emergencies; hence, it does not warrant the absence of RSI. Nowadays, there are emerging technologies to prevent RSI; instead of conventional counting techniques, electronic devices that accurately track the count of surgical items and identify if there are any missing items by radiofrequency. These technologies are still under development and are considered relatively expensive^17–19^.

**Table 1:** RSI-associated complications

Early complications	Late complications
Pain	Adhesions
Infection	Fistula formation
Hemorrhage	Abscess formation
Obstruction	Bowel perforation
Peritonitis	Sepsis
Organ damage	Chronic pain
Nausea and vomiting	Nutritional deficiencies
Wound dehiscence	
Delayed healing	

## Conclusion

Each case of Gossypiboma is unique, and each case report can provide valuable insights into the specific circumstances, symptoms, and management options involved. Therefore, it should be kept in mind and be considered in any patients that present with new abdominal symptoms following a history of the recent previous procedure. Since RSI is a preventable iatrogenic complication that can have detrimental effects on both patients and physician , every proactive step should be made to prevent and minimize the morbidity-related complications.

Learning points and recommendations

- Prevention is key: The best way to avoid gossypibomas is to prevent them from happening in the first place. Surgical teams, including surgeons and assistants, should check the number of instruments and cotton towels before and at the end of the operation before closing the wound.
- Communication is critical. Effective communication among surgical team members is vital to prevent gossypibomas. Surgeons, nurses, and other team members should have clear lines of communication and be encouraged to speak up if they notice any discrepancies or concerns.
- Proper documentation is essential: Surgeons and nurses should ensure that all sponges are correctly counted and documented, and any discrepancies should be resolved before the patient leaves the operating room.

## Ethical approval

IRB approval.

## Consent

Written informed consent was obtained from the patient for the publication of this case report and accompanying images. A copy of the written consent is available for review by the Editor-in-Chief of this journal on request.

## Sources of funding

NA.

## Author contribution

M.A.A.: operating surgeon, wrote the discussion section; H.S.A.: assisting surgeon wrote the abstract; S.A.A.: data collection, searched for reference; M.A.F.: data collection, searched for reference; S.M.A.: wrote the highlights, introduction, learning points, and recommendation and revised the final manuscript.

## Conflicts of interest disclosure

The authors declare that they have no financial conflict of interest with regard to the content of this report.

## Research registration unique identifying number (UIN)

1. Name of the registry: NA.
2. Unique Identifying number or registration ID: NA.
3. Hyperlink to your specific registration (must be publicly accessible and will be checked): NA.

## Guarantor

Mohammed Alsuhaimi.

## Data availability statement

Not applicable.

## Provenance and peer review

Not commissioned, externally peer-reviewed.
